# The clinical development of sGC modulators, riociguat and vericiguat

**DOI:** 10.1186/2050-6511-16-S1-A2

**Published:** 2015-09-02

**Authors:** Frank Misselwitz

**Affiliations:** 1 Global Development Cardiovascular, Bayer Pharma AG, 42113 Wuppertal, Germany

## Clinical background

The NO – sGC – cyclic GMP pathway is a key pathway to regulate vascular tone. Its stimulation leads to vasodilatation; NO deficiency leads to vasoconstriction, inflammation and fibrotic remodelling. Riociguat and vericiguat are two representatives of modulators of the soluble guanylate cyclase and are able to increase cyclic GMP even in conditions of NO deficiency.

Riociguat, a first lead sGC stimulator, was developed in two different forms of pulmonary hypertension (PH). PH is a rare, severe disease which untreated has a high morbidity and mortality. It is characterized by endothelial dysfunction, NO deficiency, vascular remodelling and results and devastating increases of the mean pulmonary artery pressure above 25 mm Hg. This condition leads to right heart failure, is associated with impaired exercise capacity and death. Riociguat has been successfully developed in two different forms of PH, pulmonary arterial hypertension (PAH, WHO group 1), and chronic thromboembolic pulmonary hypertension (CTEPH, WHO group 4). It was shown to significantly improve exercise capacity, time to clinical worsening, and also led to meaningful hemodynamic improvements. Riociguat (Trade name: Adempas) is now the first approved medical treatment for patients with CTEPH who are not eligible to undergo surgery, or who have residual disease after pulmonary endarterectomy. Riociguat's ongoing clinical development program includes PH-IIP and diffuse cutaneous sclerodermia, as well as an exploratory study in cystic fibrosis.

Vericiguat is currently in late stage clinical development for the treatment of heart failure with reduced and also with preserved ejection fraction (HFrEF and HFpEF). Data from the LEPHT trial with riociguat in patients with PH due to left heart disease hint to the usefulness of sGC modulation in patients with heart failure. The SOCRATRES program studies the efficacy and safety of vericiguat in patients with HFrEF and HFpEF and assesses its effect on ventricular decongestion, as measured by the biomarker NT-pro BNP, and left atrial size. Results of that program will be reported later this year.

## Conclusion

With Riociguat (Adempas) and Vericiguat Bayer is developing two representatives of the new class of sGC modulators in a large variety of clinical indications and conditions.

